# Oral health literacy and oral health-related quality of life among inpatients: the mediating effects of oral health-related self-efficacy: a cross-sectional study

**DOI:** 10.1186/s12903-025-06436-x

**Published:** 2025-07-02

**Authors:** Hang Li, Yu Zhang, Qiying He, Peng Gu, Qin Fu, Dandan Xie

**Affiliations:** 1 Department of Urology, Institute of Urology, West China Hospital, West China School of Nursing, Sichuan Clinical Research Center for kidney, Sichuan University, Chengdu, Sichuan 610041 China; 2 Clinic Medical College, Dental Department, Affiliated Hospital of Chengdu University, Chengdu, 610081 China

**Keywords:** Inpatients, Oral health literacy, Oral-related self-efficacy, Oral health-related quality of life, Mediating effect

## Abstract

**Background:**

Oral diseases can affect organs throughout the body. A healthy oral condition is an important part of maintaining overall health. Oral health literacy and self-efficacy contribute to promoting oral health. However, few studies have reported the oral health literacy and oral health-related self-efficacy of inpatients and the influence mechanism on the oral health-related quality of life is also unclear.

**Aims:**

To explore the mediating effect of oral health-related self-efficacy (OHRSE) of inpatients on oral health literacy (OHL) and oral health-related quality of life (OHRQoL).

**Methods:**

In January 2025, a cross-sectional survey was conducted among 449 inpatients in 10 public hospitals in eastern Sichuan (Guang ‘an and Dazhou), western Sichuan (Chengdu and Deyang), southern Sichuan (Neijiang, Leshan, Panzhihu and Zigong), and northern Sichuan (Guangyuan and Nanchong) using the Short Form of Health Literacy Dental Scale (HeLD-14), the Geriatric Self-Efficacy Scale for Oral Health (GSEOH) and the Geriatric Oral Health Assessment Index (GOHAI). T-test and ANOVA were used for inter-group comparison, Pearson correlation analysis was used to explore the relationship among OHL, OHRSE and OHRQoL, and linear regression and Model 4 of Process v4.2 macro program were used to conduct significance test on the mediating effect of OHRSE between inpatients’ OHL and OHRQoL.

**Results:**

Among 449 inpatients,71.8% were female, and 80.3% of the inpatients reported no oral related diseases. The scores of their OHL, OHRSE and OHRQoL were 51.14 ± 9.97, 58.67 ± 10.27 and 49.60 ± 9.61 respectively. The Pearson correlation coefficients of patients’ OHL andOHRSE, OHL and OHRQoL, OHRSE and OHRQoL are 0.608, 0.383, 0.383, and *p* < 0.001. OHRSE was the mediating variable of OHL and OHRQoL, the direct effect (β = 0.229, 95% CI: 0.127–0.331, *p* < 0.001) and the mediating effect (β = 0.140, 95% CI: 0.074–0.217, *p* < 0.001) accounted for 62.15% and 37.85% of the total effect (β = 0.369, 95% CI: 0.286–0.451, *p* < 0.001), respectively.

**Conclusion:**

Medical institutions and medical staff can promote inpatients’ oral health-related quality of life by taking measures to improve the inpatients’ oral health literacy or enhance their oral - related self - efficacy.

**Supplementary Information:**

The online version contains supplementary material available at 10.1186/s12903-025-06436-x.

## Introduction

As one of the most common non - communicable diseases, oral diseases affect the oral health of 3.5 billion people throughout the life cycle, from childhood to old age [1]. Oral diseases have a potential impact on multiple organs of the whole body. Good oral health is the starting point for overall physical health and well - being [2, 3]. Oral health is defined as a state free from chronic oral and facial pain, oral and throat cancer, oral infections and ulcers, periodontal (gum) diseases, tooth decay, tooth loss, and other diseases and conditions that limit an individual’s ability to bite, chew, smile, speak, and maintain social and mental health [4]. Strengthening oral health work and improving the level of oral health have become a consensus of society and the country. Previous reports have shown that medications used during hospitalization can cause various oral diseases such as ulcers and infections, and changes in oral health can affect the treatment outcomes of overall health [5]. Currently, the oral health management of most inpatients is usually carried out by nurses [6]. Although most of them recognize the importance of oral health management, factors such as insufficient resources, barriers in oral care practice and knowledge, and nurse-related obstacles have an impact on the management of oral health [7]. Therefore, the importance of paying attention to the oral health-related issues of inpatients is self-evident.

Oral health literacy (OHL) refers to the ability of individuals to obtain, process, and understand basic health information and services and make appropriate oral health decisions [8], including the ability to read and understand written texts, effectively communicate health-related information, use the healthcare system, and achieve and maintain physical health [9]. Previous studies have confirmed the correlation between OHL and various oral health indicators and oral health behaviors [10], patients with low OHL tend to have a poor quality of life related to oral health [11], improving OHL can affect oral health by enhancing patients’ treatment compliance and self-management skills [9]. The self-efficacy is a person’s confidence in their ability to undertake behaviors that may lead to expected health outcomes. Oral health-related self-efficacy (OHRSE) refers to an individual’s subjective awareness or judgment of whether they can effectively protect their oral health [12, 13]. People with high self-efficacy tend to have received a higher level of education and are more confident in behaviors and abilities such as using toothbrushes and dental floss to maintain oral hygiene [14]. Therefore, the better self-efficacy of patients, the better quality of life of oral health [15].

Studies have shown that enhancing self-efficacy can help improve health literacy levels, thereby promoting people to adopt healthy behaviors and lifestyles [16]. However, some studies also suggest that people with high self-efficacy have better health literacy, stronger self-management behaviors and more confidence [17]. Overall, the exploration of the relationship among OHL, OHRSE and OHRQoL in the existing literature needs to be further confirmed. This study aims to investigate the current status of oral health literacy, oral health-related self-efficacy and oral health-related quality of life among inpatients, and further reveal the relationship among the above three variables. Our research hypothesis is that oral health-related self-efficacy has a mediating effect in oral health literacy and oral health quality of life.

## Methods

### Study design

A cross - sectional study design was adopted. Using the convenient sampling, a total of 10 public hospitals were selected in eastern Sichuan (Guang ‘an and Dazhou), western Sichuan (Chengdu and Deyang), southern Sichuan (Neijiang, Leshan, Panzhihua and Zigong), and northern Sichuan (Guangyuan and Nanchong) to collect information related to the oral health status of inpatients from January 2nd to January 6th, 2025. The article reports in accordance with Strobe (see the Additional file 1).

### Inclusion and exclusion criteria 

Patient inclusion criteria: (1) More than 18 years old; (2) Hospitalization duration more than 24 h; (3) Willing to participate in this study voluntarily. Exclusion criteria: Patients with cognitive or mental disorders, or those with reading disabilities.

### Instruments 

The questionnaire in this study consists of 4 parts: general information, patients’ oral health literacy, oral health-related self-efficacy, and oral health assessment index (See the Additional file 2).

#### General information 

The general information part was designed by the researchers based on literature review. It includes 9 items: sex, age, educational, household monthly income, spouse, whether suffered from oral diseases, whether exist oral health related medical experience, brushing frequency, and whether exist dental prostheses, that means patients whether exist the prostheses fabricated after partial or total loss of the upper and lower jaw teeth.

#### Oral health literacy (OHL) 

The “Short Form of Health Literacy Dental Scale (HeLD-14)” translated into Chinese by Yan was adopted [18]. This scale is used to investigate the ability of patients to obtain, process, and understand basic oral health information and required services in order to make appropriate health decisions. It includes 14 items in 7 dimensions: receptivity, understanding, support, financial burden, medical treatment, communication, and application. The scale uses a 5-point Likert scale, where 1 point represents “very difficult” and 5 points represent “no difficulty at all”. The higher the total score, the better the health literacy. The Cronbach’s α coefficient of this scale is 0.908, and the test-retest reliability is 0.988.

#### Oral health-related self-efficacy (OHRSE) 

The “Geriatric Self-Efficacy Scale for Oral Health (GSEOH)” has been translated into Chinese by Xu and was subsequently employed in this study [13]. This scale is used to detect patients’ beliefs in their abilities to achieve the goal of oral health. It includes 20 items in 3 dimensions: oral hygiene habits, oral function, and dental visit habits. The Likert 4-point scoring method is adopted, where 1 point represents “completely unconfident” and 4 points represent “very confident”. The higher the total score, the higher the self-efficacy level. The Cronbach’s α coefficient of this scale is 0.913, and the test-retest reliability coefficient is 0.743.

#### Oral health assessment index 

The “Geriatric Oral Health Assessment Index (GOHAI)” has been translated into Chinese by Wang and was utilized in this study [19]. This scale is used to detect the quality of patients’ OHRQoL. It includes 12 items in 3 dimensions: functional limitations, psychological discomfort, and pain discomfort. The Likert 5-point scoring method is used, where 1 point represents “very often” and 5 points represent “never”. The higher the total score, the better the oral health-related quality of life. The Cronbach’s α coefficient of this scale is 0.81, and the test-retest reliability is 0.866.

### Sample size 

According to the sample size formula N=$\:\frac{{U}_{1-\alpha\:/2}^{2}*{S}^{2}}{{d}^{2}}$,combined with the literature, the mean score of OHL is taken as 6.29 [20], the mean score of OHRSE is taken as 9.8 [13], and the mean value of the GOHAI is taken as 8.49 [21]. The allowable error was 1 and α was 0.05. The calculated sample sizes are 152, 369, and 277 respectively. Taking the largest sample size of 369 and calculating with a 20% non - response rate, the final sample size should include at least 443 patients.

### Data collection 

Prior to questionnaire distribution, survey investigator at each participating hospital received training on the study’s objectives and methods, along with a detailed explanation of each questionnaire item. After fully obtaining the informed consent of the hospitalized patients, the patients were asked to fill in the questionnaire through the Wenjuan star (An online survey platform in China). The investigator would provide explanations for the patients who were confused, so as to ensure that the patients can answer accurately according to their actual situations. The time taken to complete the questionnaire was within 10–30 min.

### Statistical analysis 

Microsoft Office Excel 2021 and IBM SPSS Statistics for Windows, Version 25.0 were used to establish and process the data. The basic characteristics of patients are described by adoption rate and percentage. The OHL, OHRSE and the OHRQoL that conform to the normal distribution are described by mean ± standard deviation. Pearson correlation analysis was employed to explore the relationships among OHL, OHRSE, and the OHRQoL. Based on the homogeneity of variance, the t - test and analysis of variance are used to analyze the differences in the health - related quality of life among different patients. The Welch ANOVA is used for the OHRQoL of patients with different tooth - brushing frequencies. Linear regression was adopted to explore the predictive effect of OHRSE on OHL and OHRQoL. The Model 4 of the Process v4.2 macro program was used to conduct a significance test on the mediating effect of OHRSE between OHL and OHRQoL of inpatients. A resampling of 5000 times was required to estimate the mediating effect, with 95% significance in Bootstrap. If the interval did not include 0, it indicated that the corresponding effect value was statistically significant. A difference with *p* < 0.05 was considered statistically significant.

### Ethics 

This study has been approved by the Biomedical Ethics Committee, West China Hospital, Sichuan University (Approval No. [967] in 2024). And all the participants provided informed consent for this study. The study was conducted in strict accordance with the Declaration of Helsinki [22].

## Results 

449 inpatients completed the questionnaires in total, and the response rate is 100%.

### General information 

323 are female among 449 inpatients, accounting for 71.8% of the total number. 81.3% of the patients self-reported no oral diseases, but 53.7% of the patients had oral medical experience, as shown in Table 1.

**Table 1:** General information (*N* = 449)

Variables	*n*(%)
Age	
＜30	144(32.1)
30-44	177(39.4)
≥45	128(28.5)
Education	
Junior high school or below	64(14.3)
High school/Junior college	117(26.0)
Bachelor degree or above	268(59.7)
Is there a spouse	
Yse	318(70.8）
No	131(29.2)
Household monthly income	
≤6000	117(26.1)
6001-10000	125(27.8)
≥10001	207(46.1)
Whether suffered from oral disease	
Yse	84(18.7)
No	365(81.3)
Whether exist oral health related medical experience	
Yes	241(53.7)
No	208(46.3)
Whether exist dental prostheses	
Complete denture	24(5.3)
Partial denture	109(24.3)
Without denture	316(70.4)
Brushing frequency (times/d)	
0	6(1.3)
1	120(26.7)
2	323(72.0)

### Correlation analysis 

The scores of OHL, OHRSE and OHRQoL of 449 patients were 51.14 ± 9.97, 58.67 ± 10.27, and 49.60 ± 9.61 respectively. The results of Pearson correlation analysis showed that there was a pairwise positive correlation among the inpatients’ OHL, OHRSE and OHRQoL and *p* < 0.001, as shown in Table 2.

**Table 2:** The relevance between OHL, OHRSE and OHROoL (*N* = 449)

Variables	OHL	OHRSE	OHRQoL
OHL	-	0.608^**^	0.383^**^
OHRSE	0.608^**^	-	0.383^**^
OHRQoL	0.383^**^	0.383^**^	-

Note: “**” represents *p*<0.001

### Discrepancy of OHRQoL among inpatients 

T-test and ANOVA results showed that there were statistical differences in gender of inpatients, whether suffered from oral diseases and whether existed dental prostheses, as shown in Table 3.

**Table 3:** Discrepancy of OHRQoL among inpatients (*N* = 449)

Variables	OHRQoL	t/F	*P*
Sex		2.162	0.031
Male	51.17±9.23		
Female	48.99±9.70		
Age		0.466	0.628
<30	52.20±9.47		
35-45	49.16±9.98		
≥45	49.54±9.27		
Education		1.159	0.329
Junior high school or below	49.75±10.39		
High school/Junior college	49.97±9.51		
Bachelor degree or above	49.41±9.48		
Is there a spouse		1.167	0.244
Yes	49.26±9.50		
No	50.43±9.85		
Household monthly income		0.813	0.444
≤6000	48.63±10.76		
6001-10000	52.02±9.18		
≥10001	49.90±9.17		
Whether suffered from oral disease		2.685	0.08
Yes	47.08±9.91		
No	50.18±9.45		
Whether exist oral health related medical experience		3.753	<0.001
Yes	48.05±9.47		
No	51.41±9.46		
Whether exist dental prostheses		17.849	<0.001
Complete denture	50.08±10.83		
Partial denture	45.01±9.39		
Without denture	51.15±9.10		
Brushing frequency (times/d)		13.109	0.957
0	52.00±19.60		
1	49.6±9.40		
2	49.56±9.47		

Note: The scores of OHRQoL are described using the mean ± standard deviation

### Mediating effect analysis 

The mediating effect of OHRSE between the OHL and OHRQoL of inpatients was explored using linear regression. The variables with *p* < 0.1 in the univariate analysis results, such as age, whether suffered from oral diseases, whether exist dental prostheses, and the oral health related medical experience, were included in the model, as shown in Table 4, and VIF of these variables were below 5. Model 1 (R^2^ = 0.399, F = 58.88, *p* < 0.001) showed that the control variable of the oral health-related medical experience significantly affected the OHRSE of inpatients (*p* < 0.01). Model 2 (R^2^ = 0.252, F = 29.78, *p* < 0.001) showed that the control variables of sex, the oral health-related medical experience, and whether exist dental prostheses significantly affected the OHRQoL of inpatients (*p* < 0.001). Model 3 (R^2^ = 0.269, F = 27.11, *p* < 0.001) showed that under the significant influence of the mediating variable of OHRSE (β = 0.159, *p* < 0.01), the independent variable of OHL significantly affected the OHRQoL (β = 0.302, *p* < 0.001).

**Table 4:** The mediating effect of OHRSE on OHL and OHRQoL

Variables	B	t	*p*	95%CI (LL, UL)
Model 1				
Constant	27.861	13.425	<0.001	23.782, 31.940
Sex	-0.772	-0.868	0.386	-2.521, 0.977
Suffered from oral disease	-1.948	-1.903	0.058	-3.960, 0.064
Oral health related medical experience	-2.571	-3.198	0.001	-4.151, -0.991
Dental prostheses	-0.384	-0.560	0.576	-1.733, 0.964
OHL	0.650	16.660	<0.001	0.573, 0.727
R^2^		0.399		
Adjust R^2^		0.392		
F		58.877^***^		
Model 2				
Constant	34.818	16.072	<0.001	30.561, 39.076
Sex	-4.105	-4.419	<0.001	-5.931, -2.280
Suffered from oral disease	-1.908	-1.785	0.075	-4.008, 0.193
Oral health related medical experience	-3.201	-3.815	<0.001	-4.851, -1.552
Dental prostheses	-2.722	-3.800	<0.001	-4.129, -1.314
OHL	0.406	9.968	<0.001	0.326, 0.486
R^2^		0.252		
Adjust R^2^		0.243		
F		29.779^***^		
Model 3				
Constant	30.380	11.950	<0.001	25.384, 35.377
Sex	-3.982	-4.329	<0.001	-5.790, -2.175
Suffered from oral disease	-1.597	-1.505	0.133	-3.684, 0.489
Oral health related medical experience	-2.792	-3.324	0.001	-4.443, -1.141
Dental prostheses	-2.660	-3.753	<0.001	-4.054, -1.267
OHL	0.302	5.885	<0.001	0.201, 0.403
OHRSE	0.159	3.246	0.001	0.063, 0.256
R^2^		0.269		
Adjust R^2^		0.259		
F		27.106^***^		

Note: Model 1 represents OHL + control variables → OHRSE; Model 2 represents OHL + control variables → OHRQoL; Model 3 represents OHL + OHRSE + control variables → OHRQoL; “***” represents *p* < 0.001

**Fig. 1:**
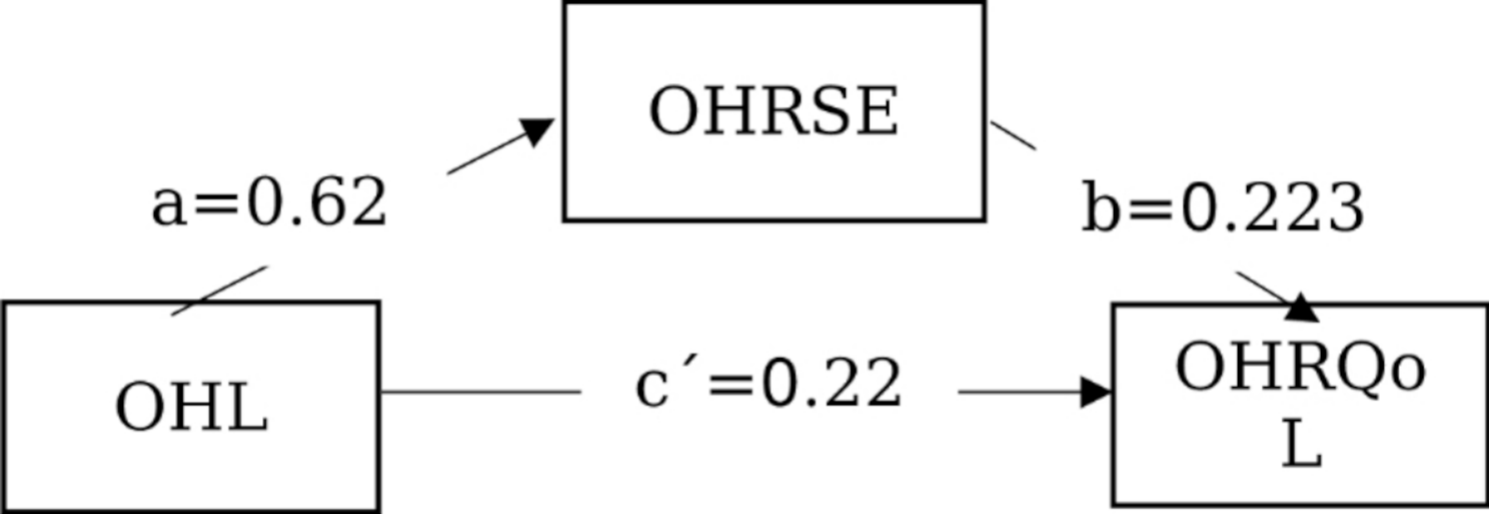
The mediating effect of OHRSE on OHL and OHRQoL. Note: “a” presents coefficient of OHL→OHRSE; “b” presents coefficient of OHRSE→OHRQoL; “c´”presents coefficient of OHL→OHRQoL or the direct effect of OHL→OHRQoL; “c” presents the total effect of OHL→OHRQoL; “***” represents p < 0.001

The Bootstrap method was used to verify the mediating effect, and the results showed that the direct effect (β = 0.229, 95% CI: 0.127–0.331, *p* < 0.001) and the mediating effect (β = 0.140, 95% CI: 0.074–0.217, *p* < 0.001) accounted for 62.15% and 37.85% of the total effect (β = 0.369, 95% CI: 0.286–0.451, *p* < 0.001), respectively. The mediating effect model is show in Fig. 1.

## Discussion

Understanding the OHL, OHRSE and OHRQoL of inpatients can help provide clinical evidence for medical staff to optimize oral health-related services. This study investigated the OHL, OHRSE and OHRQoL of inpatients, and found that the OHL, OHRSE and OHRQoL of inpatients were all at a medium level, and the OHRSE was an intermediate variable between OHL and OHRQoL.

The oral health literacy of inpatients in this study was lower than that of middle-aged and elderly patients with dentition defects investigated by Zhang [20]. The reason might be related to the fact that the previous study population consisted of patients in stomatological hospitals who could be influenced by more professional oral health-related information. Cao reported that the oral health knowledge, attitude and behavioral practice of the community population were related to frailty [23]. This indicates that there is still much room for improvement in the OHL of ordinary inpatients. For inpatients with low-level OHL who may have major oral health problems, appropriate oral health-related treatment and education may help reduce the risk of other system health conditions being threatened by oral health problems.

There is a lack of evidence in domestic and foreign literature regarding the OHRSE of inpatients. A study on the OHRSE of community older people in Luzhou, Sichuan Province, showed that the OHRSE of this population was at a medium level [24], slightly lower than the results of this survey. The reason is related to the fact that community elderly people with older age and lower education level may be more lacking in confidence in their ability to achieve the goal of oral health [25].

The OHRQoL of inpatients in this study was higher than the 45.62 ± 8.49 of the community elderly population surveyed by Yu conducted the research in Shijiazhuang [21], but lower than the 53.79 ± 11.17 of the community population surveyed by He in Haikou [26]. This difference can be explained by age factors as well as different oral health policies and economic development among regions. Similar to OHRSE, the OHRQoL of inpatients is also not fully reported. Based on the results of this survey, the oral health status of inpatients still needs to be further improved, which also provides an evidence basis for health departments and medical institutions to formulate or provide oral-related health services.

Previous studies have shown that poor oral health status upon admission may prolong the length of hospital stay of patients, and providing or strengthening oral care during the recovery period in hospital can help promote the functional recovery of patients and improve malnutrition [27]. Therefore, it is of great significance to carry out relevant oral medical and nursing services for patients with poor oral health status during their hospital stay. OHL may be one of the ways to improve the quality of life related to oral health of bladder cancer patients [28]. Patients with a high level of OHL are associated with the use of preventive dental care [29]. They are better at collecting and processing health information to support appropriate health decisions [24], and then take correct daily behaviors to promote oral health, such as brushing skills and maintaining personal oral hygiene [30]. OHRSE plays a mediating role between OHL and OHRQoL. Existing studies have confirmed that knowledge can indirectly affect practice through attitude, and attitude affects self-efficacy through practice, thereby improving the OHRQoL [31]. People with OHL, due to their strong confidence in maintaining oral health, are more likely to adopt healthy behaviors, such as establishing good brushing habits and reasonable dietary strategies. These behaviors help reduce dental plaque and bleeding and lower the risk of long-term periodontal treatment [32, 33].

This study revealed that the OHL, OHRSE, and OHRQoL among inpatients remain suboptimal and warrant enhancement. By elucidating the mediating role of OHRSE between OHL and OHRQoL, these findings underscore the imperative of prioritizing interventions targeting inpatients’ OHL and OHRSE. Furthermore, this research offers a robust clinical framework and strategic direction for advancing oral health management among hospitalized patients. Medical staff or medical institutions can organize training sessions or lectures on oral health knowledge and behaviors for inpatients to enhance their understanding and awareness of oral health information. Meanwhile, intervention programs can be implemented to target and increase the confidence and ability to perform certain specific oral tasks, such as increasing the frequency of brushing teeth, so as to enhance the inpatients’ OHRSE and thus improve the OHRQoL.

## Limitation

The study has some limitations: the cross-sectional survey may lack persuasiveness in clarifying causal relationships, and longitudinal research design could strengthen result reliability in future studies. The use of convenient sampling only among inpatients limited the external validity and universality of the research results, and the data are from patients’ self-reports, they may be deviated by factors like patient recall and social expectations. Notably, this study has its merits. It investigated inpatients from 10 hospitals and expanded the sample size during calculation to ensure representativeness. The research model’s accuracy might be affected by not including confounding variables such as inpatients’ health status, oral medical service utilization, and oral care support. Future research should consider these variables to refine the model and improve conclusion reliability and generalizability.

## Conclusion

This study shows that the oral health literacy, oral health-related self-efficacy and oral health-related quality of life of current clinical inpatients still need to be further improved. Medical institutions and medical staff providing or strengthening the oral health literacy and oral health-related self-efficacy of inpatients during the clinical treatment and care process can help provide paths and opportunities for inpatients to improve their oral health status.

## Electronic supplementary material

Below is the link to the electronic supplementary material.

Supplementary Material 1

Supplementary Material 2

## Data Availability

The data cannot be made public for privacy reasons. A reasonable request to reuse the data can be submitted to the corresponding author.

## Abbreviations

OHL: Oral Health Literacy
OHRSE: Oral Health-related Self-efficacy
OHRQoL: Oral Health-related Quality of Life
HeLD-14: Short Form of Health Literacy Dental Scale
GSEOH: Geriatric Self-Efficacy Scale for Oral Health
GOHAI: Geriatric Oral Health Assessment Index
