# Supplementary material for: Oral health literacy and oral health-related quality of life among inpatients: the mediating effects of oral health-related self-efficacy: a cross-sectional study
Source: BMC Oral Health. 2025 Jul 2;25:1049. doi: 10.1186/s12903-025-06436-x (PMC12225096; doi:10.1186/s12903-025-06436-x)
Supplement: Supplementary file 1 — Supplementary Material 1 [file 12903_2025_6436_MOESM1_ESM.docx]

**Questionnaire on Oral Health Status of Inpatients**

Dear sir/madam,

In recent years, the increasing incidence of oral diseases not only affects patients' eating and nutrition intake, but also the changes in patients' facial structure and appearance caused by it will increase patients' psychological burden. Oral health is the foundation of overall health and has attracted great attention from the country and society. Understanding your current oral health status will help medical and nursing staff provide you with better oral care advice and measures to protect your oral health. Therefore, we sincerely hope that you will participate in and complete this survey. Please fill in the questionnaire truthfully according to your actual situation. Your information will only be used for the collection of data on this oral health status and will not be disclosed. Please rest assured. Thank you again for your support and cooperation!

**Ⅰ General Information**

1.Gender:

Male Female

2. Age: ___ years old

3. Education Level:

Junior high school and below High school/ junior college

Bachelor degree or above

1. Do you have a spouse?

Yes No

1. Monthly household income:

≤6000 6001 - 10000 ≥10001

1. Do you exist false tooth:

Complete dentures Partial dentures None

1. How often do you brushing per day:

0 1 2

1. Did you suffer from any oral diseases?

Yes No

1. Have you ever had an oral health-related medical experience?

Yes No

**II Oral Health Status Survey**

(I) Simplified Oral Health Literacy Scale (HeLD-14)

This scale is used to investigate your personal ability to obtain, process and understand basic oral health information and the required services to make appropriate health decisions.

1. You pay attention to your teeth or oral health.

Very difficult Difficult Average Not too difficult No difficulty at all

2. You spend time doing things that are beneficial to your teeth or oral health.

Very difficult Difficult Average Not too difficult No difficulty at all

3. You can understand relevant written information, such as the medical order given to you by your dentist.

Very difficult Difficult Average Not too difficult No difficulty at all

4. You can understand the information brochures on teeth or oral health provided in dental clinics and waiting rooms.

Very difficult Difficult Average Not too difficult No difficulty at all

5. You can take your family members or friends to see a dentist.

Very difficult Difficult Average Not too difficult No difficulty at all

6. When you go to see a dentist, you will ask someone else to go with you.

Very difficult Difficult Average Not too difficult No difficulty at all

7. You can afford the cost of seeing a dentist (treatment and examination fees).

Very difficult Difficult Average Not too difficult No difficulty at all

8. You can afford the cost of your teeth or oral care.

Very difficult Difficult Average Not too difficult No difficulty at all

9. You know how to see a dentist.

Very difficult Difficult Average Not too difficult No difficulty at all

10. You know what to do when seeing a dentist.

Very difficult Difficult Average Not too difficult No difficulty at all

11. Regarding your teeth or oral care, you will seek the advice of dental professionals.

Very difficult Difficult Average Not too difficult No difficulty at all

12. Regarding your teeth or oral care, you can make corresponding decisions using the information provided by your dentist.

Very difficult Difficult Average Not too difficult No difficulty at all

13. You can follow the dentist's instructions.

Very difficult Difficult Average Not too difficult No difficulty at all

14. Regarding your teeth or oral health, you can adopt the dentist's advice and make corresponding decisions.

Very difficult Difficult Average Not too difficult No difficulty at all

(II) General Self-Efficacy Scale for Oral Health (GSEOH)

This scale is used to measure your belief in your ability to achieve the goal of oral health, including three dimensions: oral hygiene habits, oral function, and oral visit habits.

1. I can check the cleanliness of my mouth.

Completely unconfident Unconfident Confident Very confident

2. I can observe the cleanliness of my tongue.

Completely unconfident Unconfident Confident Very confident

3. I brush my teeth in the correct way (such as the Bass brushing method).

Completely unconfident Unconfident Confident Very confident

4. I can brush my teeth well with a toothbrush.

Completely unconfident Unconfident Confident Very confident

5. I will rinse my mouth after each meal.

Completely unconfident Unconfident Confident Very confident

6. I will listen to and follow the necessary oral health advice.

Completely unconfident Unconfident Confident Very confident

7. Even if I'm very busy, I will stick to oral care.

Completely unconfident Unconfident Confident Very confident

8. I can maintain my oral hygiene.

Completely unconfident Unconfident Confident Very confident

9. I can speak easily even when my mouth is dry.

Completely unconfident Unconfident Confident Very confident

10. I can swallow easily even without drinks or soup.

Completely unconfident Unconfident Confident Very confident

11. I can chew any food.

Completely unconfident Unconfident Confident Very confident

12. I can enjoy the pleasure of eating.

Completely unconfident Unconfident Confident Very confident

13. I can communicate with others without worrying about my oral problems (bad breath, dental aesthetics, tooth loss, etc.).

Completely unconfident Unconfident Confident Very confident

14. I can talk fluently.

Completely unconfident Unconfident Confident Very confident

15. I can recover quickly after my mouth or teeth feel uncomfortable.

Completely unconfident Unconfident Confident Very confident

16. Even if I have oral problems, they do not affect my daily life.

Completely unconfident Unconfident Confident Very confident

17. I'm very confident about my mouth.

Completely unconfident Unconfident Confident Very confident

18. Even after the treatment is over, I will continue to go to the clinic regularly to prevent recurrence.

Completely unconfident Unconfident Confident Very confident

19. I will go for regular oral health checks.

Completely unconfident Unconfident Confident Very confident

20. Even when I'm busy, I will go for regular oral checks.

Completely unconfident Unconfident Confident Very confident

(III) Geriatric Oral Health Assessment Index (GOHAI Chinese Version)

This scale is used to measure your quality of life related to oral health, including three dimensions: functional limitations, psychological discomfort and pain discomfort.

1. Do you often limit the types and amounts of food you eat because of your teeth or dentures?

Very often Often Sometimes Seldom Never

2. Do you have difficulty biting or chewing food?

Very often Often Sometimes Seldom Never

3. Do you often feel uncomfortable or have difficulty swallowing food?

Very often Often Sometimes Seldom Never

4. Do your teeth or dentures interfere with your speech?

Very often Often Sometimes Seldom Never

5. Do you often feel uncomfortable in your mouth when eating?

Very often Often Sometimes Seldom Never

6. Do you often limit your interactions with others because of your teeth or dentures?

Very often Often Sometimes Seldom Never

7. Do you often feel dissatisfied or unhappy with the appearance of your teeth, gums or dentures?

Very often Often Sometimes Seldom Never

8. Do you often use medicine to relieve oral pain or discomfort?

Very often Often Sometimes Seldom Never

9. Do you often worry about or pay attention to problems with your teeth, gums or dentures?

Very often Often Sometimes Seldom Never

10. Do you often feel nervous or uncomfortable in front of others because of problems with your teeth, gums or dentures?

Very often Often Sometimes Seldom Never

11. Do you often feel uncomfortable when eating in front of others because of your teeth or dentures?

Very often Often Sometimes Seldom Never

12. Are your teeth or gums sensitive to cold, hot or sweet stimuli?

Very often Often Sometimes Seldom Never
